# “I Feel Contaminated in My Fake Hand”: Obsessive-Compulsive-Disorder like Disgust Sensations Arise from Dummy during Rubber Hand Illusion

**DOI:** 10.1371/journal.pone.0139159

**Published:** 2015-12-07

**Authors:** Baland Jalal, Divya Krishnakumar, Vilayanur S. Ramachandran

**Affiliations:** Center for Brain & Cognition, University of California San Diego, La Jolla, California, United States of America; University of Bologna, ITALY

## Abstract

Despite its theoretical and clinical interest, there are no experimental studies exploring obsessive-compulsive disorder (OCD)-like disgust sensations through using somatosensory illusions. Such illusions provide important clues to the nature and limits of multisensory integration and how the brain constructs body image; and may potentially inform novel therapies. One such effect is the rubber hand illusion (RHI) in which tactile sensations are referred to a rubber hand; if the experimenter simultaneously strokes a subject’s occluded hand together with a visible fake hand, the subject starts experiencing the touch sensations as arising from the dummy. In this study, we explore whether OCD-like disgust may result from contamination of a dummy hand during the RHI; suggesting a possible integration of somatosensory and limbic inputs in the construction of body image. We predicted that participants would experience sensations of disgust, when placing a disgust stimulus (fake feces, vomit or blood) on the dummy hand after establishing the RHI. We found that 9 out of 11 participants experienced greater disgust during the synchronous condition (real hidden hand and fake hand are stroked in synchrony) compared to the asynchronous control condition (real hidden hand and fake hand are stroked in asynchrony); and on average such disgust was significantly greater during the synchronous condition compared to the asynchronous control condition, *Z* = 2.7, *p* = .008. These results argue against a strictly hierarchical modular approach to brain function and suggest that a four-way multisensory interaction occurs between vision, touch, proprioception on the one hand and primal emotions like disgust on the other. These findings may inform novel clinical approaches for OCD; that is, contaminating a dummy during the RHI could possibly be used as part of an in-vivo exposure-intervention for OCD.

## Introduction

One form of obsessive-compulsive disorder (OCD) is characterized by extreme disgust and overwhelming contamination aversion. While OCD has been studied extensively using brain imaging and neurophysiology [1–4], there are few experimental studies exploring OCD-like contamination sensations through using strictly behavioral approaches. But in a recent series of experiments [5, 6], we reported some novel and counter intuitive findings. We found that when individuals with OCD traits merely observe another individual touch an object they consider “disgusting” (e.g., fake vomit or feces), it triggers their urge to cleanse their hands; and that vicarious hand cleansing (watching someone else cleansing their hands) can potentially alleviate OCD-like compulsive urges. These findings may have clinical utility for OCD; e.g., using computerized technologies (such as handheld smartphones) to conduct vicarious exposures (for details see, [6]).

The goal of the present study was to explore OCD-like contamination sensations through a well-known somatosensory illusion; with the aim of informing novel exposure approaches for OCD. Somatosensory illusions provide clues to the nature and limits of multisensory integration and how the brain constructs body image. They may also yield important clinical insights.

A striking illusion with clinical implications comes from our own group’s studies of amputees who experience phantom limbs. When amputees view the reflection of their intact arm in a mirror (creating the illusion of observing two arms), it appears to them as if the amputated arm has been resurrected. Some amputees, when observing the reflection of their intact arm being touched, report feeling touch sensations arising from their phantom limb [7]. This “mirror illusion” has since led to a novel clinical approach to relieve phantom limb pain, complex regional pain syndrome, and hemiparesis from stroke (for a review see, [8]).

The rubber hand illusion (RHI) is another such well-known effect. Botvinick and Cohen found that if they simultaneously stroked a person’s occluded a hand together with a visible rubber hand, the person starts to perceive touch sensations as arising from the dummy [9].

Our interpretation of the RHI invokes the Bayesian logic of all perceptual systems. The brain regards it as highly improbably that the random strokes and taps seen on the dummy hand are identical to what is felt on the hand simply by chance, so assumes the sensations are arising from the dummy [10–13].

There is a large body of research on the RHI and various permutations and measures of it (e.g., [12–17]). Research has also explored the RHI in the context of some psychiatric populations, such as among patients with schizophrenia and eating disorders who show a stronger malleability for the illusion [18, 19]. On the other hand, few studies have examined the direct clinical relevance of the illusion (e.g., [20]). And to our knowledge to date, no studies have examined OCD-like-contamination sensations through the RHI.

In the present study, we explored whether OCD-like disgust sensations may be triggered by contaminating a dummy hand during the RHI. We hypothesized that participants would experience feelings of contamination, when a disgust stimulus (fake feces, vomit or blood) was seen to be placed on the fake hand after establishing the illusion. If our hypothesis is supported, this procedure (i.e., contaminating a dummy limb during the RHI) could possibly be used as part of an in-vivo exposure-treatment intervention for OCD.

## Material and Methods

### Participants

Fourteen undergraduate students at the University of California, San Diego (UCSD) enrolled in the experiment. They were recruited from the Psychology Department’s human subject pool. In return for their participation, they received class credit. The participants were unaware of the experiment’s purpose. Seventy-one (10/14) percent of participants were female; participants’ ages ranged from 18–25 (*M* = 21.0, *SD* = 2.1).

### Materials and procedures

This study was approved by the institutional review board at UCSD. This study is part of a larger project at the UCSD, Center for Brain and Cognition, using strictly behavioral approaches towards developing novel exposure therapies for OCD (e.g., [5, 6]).

All participants provided written informed consent. Participants were visually presented with three disgust stimuli at a distance of approximately 20 cm. Disgust stimuli included fake: (1) feces, (2) vomit and (3) blood (made up of various substances including food items). Participants were not aware that the disgust stimuli were fake. After 10 seconds of visual exposure to the disgust stimulus, participants were asked to provide a subjective rating as to how disgusted each of the three stimulus made them feel on a 20-point Likert-scale with higher scores indicating greater disgust. The disgust stimulus (either feces, vomit or blood) that the participant rated as most disgusting was used as the participant’s disgust stimulus for the experiment (see Table 1).

**Table 1:** Participants’ disgust stimuli.

*N* = 11	*N* (%)	*M* (*SD*)
Feces	3 (27)	15.7 (5.9)
Vomit	7 (64)	16.3 (3.6)
Blood	1 (9)	17^a^
Total	11 (100)	16.2 (3.8)

Table 1 shows the number of participants who had either feces, vomit or blood as their disgust stimulus, and their mean ratings. While one participant rated “feces” and “vomit” as equally disgusting, when subsequently asked to compare, he expressed that the “feces” stimulus was more disgusting to him; so it was used as his stimulus for the experiment.

^a^ As only one participant had blood as his disgust stimulus, there is no SD to present.

Next, participants were seated upright at a table. There was a sagittal partition (approximately 45 cm X 65 cm) extending from their right collarbone onto the table. The participants placed their right arm with the palm facing down on the right side of the partition. A rubber hand was placed on the left side of the partition; a sheet of cloth was wrapped around the wrist of the rubber hand going all the way up to the shoulder, giving the illusion of a fake arm. This set-up thus occluded the participants’ real right hand and arm from view, while allowing them to observe the fake hand in a position aligned to their real hand. Participants watched continuously as the fingers of the rubber hand, and hidden real hand were simultaneously stroked in synchrony using a paintbrush. Pilot work showed that after approximately 5 minutes of such stimulation, the majority of participants would report a compelling illusion of the tactile and proprioceptive sensations arising from the rubber hand.

After continuous stroking of the rubber hand and real hidden hand for 5 minutes, a second experimenter placed the disgust stimulus on the rubber hand and a clean tissue on the real hand out of sight. If the disgust stimulus was feces or vomit, a tissue with specimen was placed on the rubber hand, while a clean tissue, slightly dampened was placed on the real hand. If the disgust stimulus was blood, a bandage with minimal blood was placed on the fake hand, while a strip of clean tissue was placed on the real hand. The clean tissue applied on the real hidden hand was thus made to mimic the feeling of having the disgust stimulus placed on the real hand. After 15 seconds of such stimulation, participants were asked to provide a subjective rating of disgust: “How disgusted do you feel?”; on a 20-point Likert-scale with higher scores indicating greater disgust.

### Clean tissue control

Immediately after participants had provided disgust ratings (see above), the disgust stimulus and the clean tissue were removed from the dummy and real hand. To sustain the illusion, the rubber hand and the real hidden hand continued to be stroked for one additional minute. Next, a clean tissue was simultaneously placed on the fake hand and real hidden hand for 15 seconds; after which the participants provided a subjective rating of disgust: “How disgusted do you feel?”; on a 20-point Likert-scale with higher scores indicating greater disgust.

The purpose of this control was to rule out the possibility that reported disgust in the experimental and control condition (i.e., when a disgust item was placed on the dummy), was due solely to physical stimulation during stroking. It may be that merely placing an object [such as a dampened tissue on the real hand (mimicking physical contact) during stroking] might elicit disgust reactions; or that experienced sensations when a clean tissue is put on the real hand might incorrectly be interpreted as disgust, once solicited by the experimenter. It also allowed for a comparison between the synchronous and asynchronous condition. If ratings were similar in the two conditions when a clean tissue was placed on the dummy and real hand, this would indicate that differences in ratings in the experimental condition versus the control condition (i.e., when a disgust item is placed on the dummy) were not due to expectation bias.

### Asynchronous control condition

The control condition was identical to the experimental condition (i.e., the synchronous condition) in all aspects except for a manipulation that diminished the vividness of the illusion; that is, touch applied to the fake hand and real hand was asynchronous. In the present study, the synchronous condition vs. the asynchronous control condition, was a within subject factor. Thus the asynchronous control condition served as a baseline providing a good control for the effects of physical stimulation and the incorporation of disgust into the body image.

### Randomization of presentation

To eliminate concerns that beginning with the RHI would create expectation bias in the disgust ratings, the order in which participants completed the experimental and control condition was randomized (see too, [13]). By randomizing the order of the conditions, one also controls for any habituation to the disgust stimulus that might occur.

### Session break

In between the two conditions, participants were asked to do imagery exercises and math exercises for approximately 2 ½ minute such that they would not accumulate the illusion in between sessions.

### Session overview

**Fig 1:**
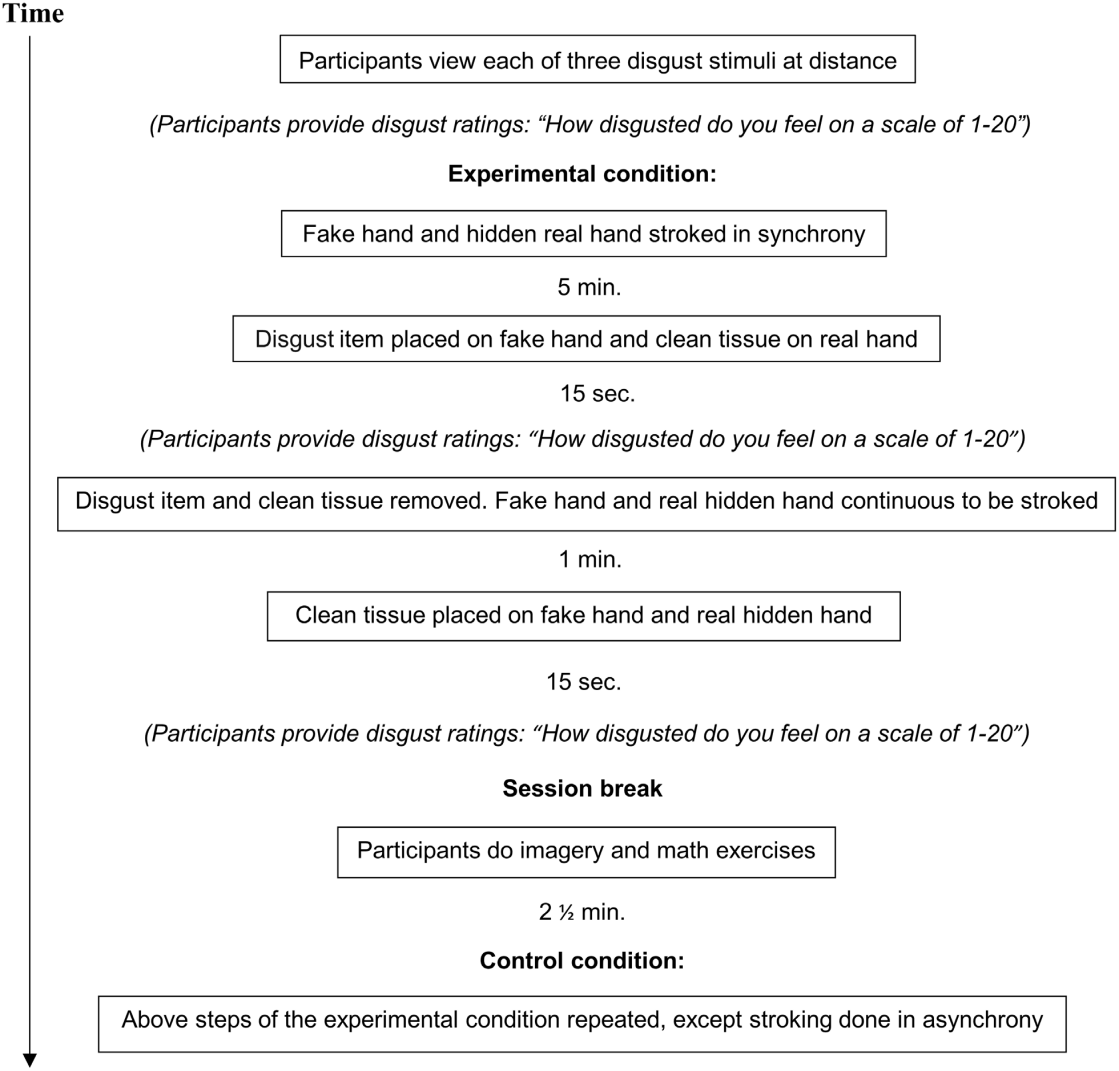
Overview of the experimental and the control condition. (A) The order in which the experimental and control condition were completed was randomized.

For an overview of the experimental and control condition, see Fig 1.

### Control experiment

To rule out the possibility that simply placing any aversive stimulus on the dummy hand during synchronous stroking (versus asynchronous stroking) would yield positive results; and to further eliminate the possibility of expectation bias, we included a control group to complete a different experiment. This control group was comprised of 18 individuals; forty-four (8/18) percent of whom were female; participants’ ages ranged from 18–25 (*M* = 21.2, *SD* = 1.6). Similar to the experimental group, participants were recruited from the department’s subject pool, and received course credit in return for their participation. They were unaware of the experiment’s purpose.

The set-up for this control was identical to the experimental set-up, with the exception that an ice cube was placed on the dummy instead of a disgust stimulus; and a rectangular shaped object with slight weight (at room temperature) was placed on the hidden real hand. Participants were asked to provide a subjective rating of “coldness”: “How cold do you feel?”; on a 20-point Likert-scale with higher scores indicating greater “coldness”.

Similar to the experimental group, participants completed one condition during synchronous stroking and another during asynchronous stroking; the order of presentation was randomized and participants were asked to do imagery and math exercises for approximately 2 ½ minute in between sessions.

### Exclusion criteria

It is well-known that not all participants experience the RHI. It was thus important to identify and exclude such participants from further analyses. Participants were instructed to give a brief verbal response as soon as they would feel (and only if they would feel) that the dummy felt as if it was their own hand (see too, [21, 22]); i.e., experienced tactile and proprioceptive sensations arising from the rubber hand. Aside from these verbal reports, we also asked participants to indicate in which condition they felt a more vivid illusion, and to what degree on a 20-point Likert-scale with higher scores indicating greater intensity of the illusion. Participants who scored less than 3 out of 20 on this “intensity of the RHI scale,” were excluded from analyses; as were those who indicated that the illusion was more vivid during the asynchronous control condition.

### Data analyses

Data were analyzed using the paired samples t-test; and for between group comparisons, the independent samples t-test. Disgust ratings during synchronous stroking did not pass the Kolmogorov–Smirnov test for normality, in which case the nonparametric Wilcoxon signed rank test and the Mann-Whitney test for between group comparisons, were used.

## Results

### Exclusion analyses

**Fig 2:**
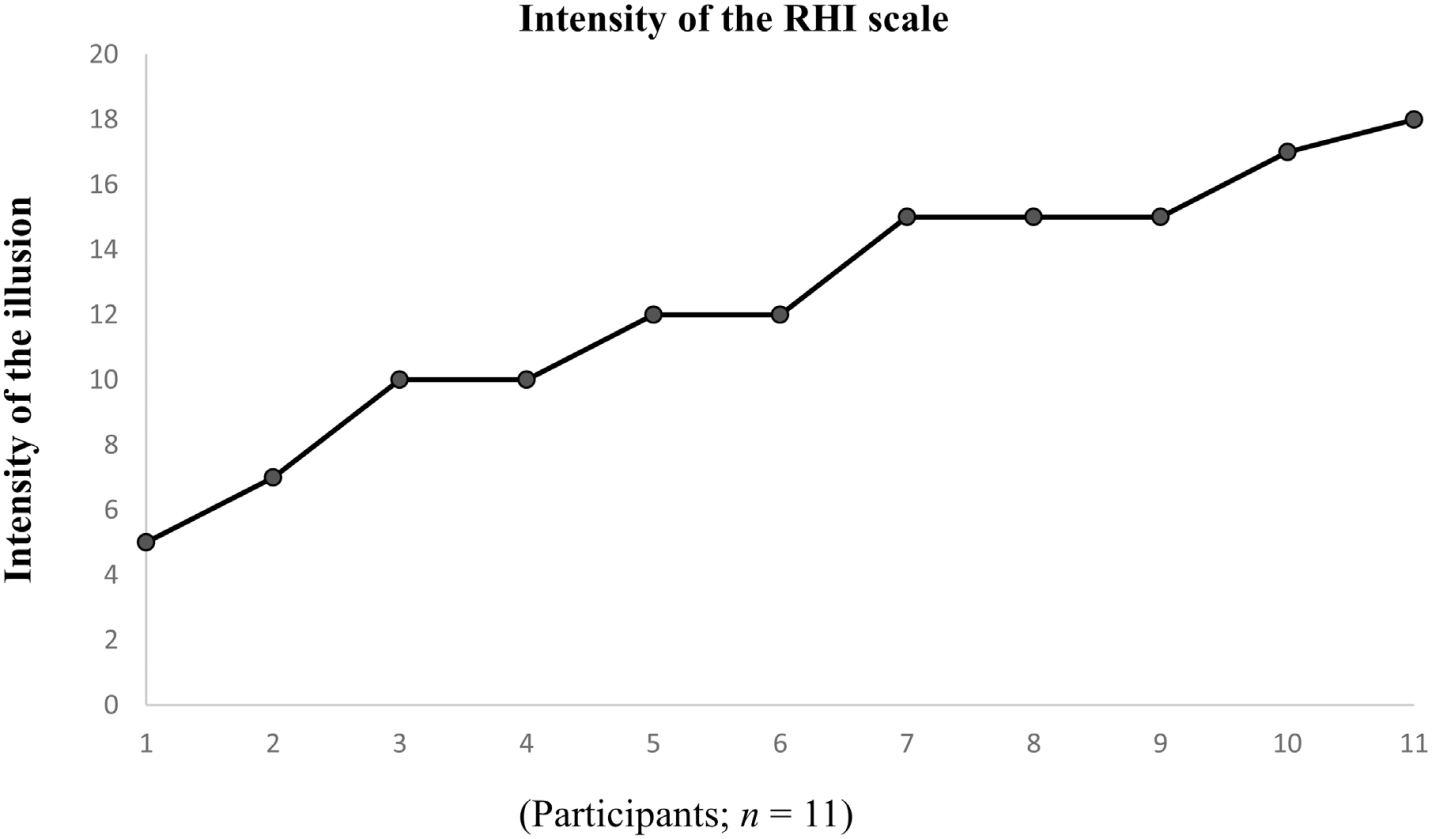
Participants’ ratings on the intensity of the RHI scale for the main experiment.

Unsurprisingly, the vast majority of participants in the experimental group experienced the RHI: 11 out of 14 (79%) reported a more intense RHI during the synchronous condition. [This result matches the usual proportions seen in classroom demonstrations and in other studies (e.g., [13])]. The intensity of the illusion ranged from 5–18 (*M* = 12.4, *SD* = 4.1), see Fig 2. Three participants were excluded: (1) one reported experiencing a more intense illusion during the asynchronous control condition; (2) another participant reported a score of 2 out of 20 on the intensity of the RHI scale during synchronous stroking (thus indicating a virtually non-existent illusion); (3) a third participant failed to provide data on the intensity of the RHI scale.

### Randomization of presentation

A Mann-Whitney test showed that during both the synchronous condition (*N* = 11, *Z* = 1.7, *p* = .08) and the asynchronous control condition (*N* = 11, *Z* = 1.6, *p* = .12), reported disgust did not differ based on order of presentation (i.e., whether the synchronous or asynchronous control condition was completed first).

### Clean tissue control

Participants reported that they did not experience any disgust sensations, when a clean tissue was simultaneously placed on the fake hand and real hidden hand during both the synchronous condition and the asynchronous condition [*M* = 1.0 (*SD* = .0) vs. *M* = 1.0 (*SD* = .0)], *N = 11*, *Z* = .00, *p* = 1.00 (with Bonferroni correction, *p* = .5). [This suggests that reported disgust in the experimental and control condition (when a disgust item was placed on the dummy) were not solely due to physical stimulation and the placing of a tissue on the real hand during stroking (i.e., ratings represent genuine disgust). It also indicates that differences in disgust ratings in the experimental versus the control condition when a disgust item was placed on the dummy, were not due to expectation bias (see materials and procedures section for details).]

### Disgust during the synchronous versus asynchronous condition

**Fig 3:**
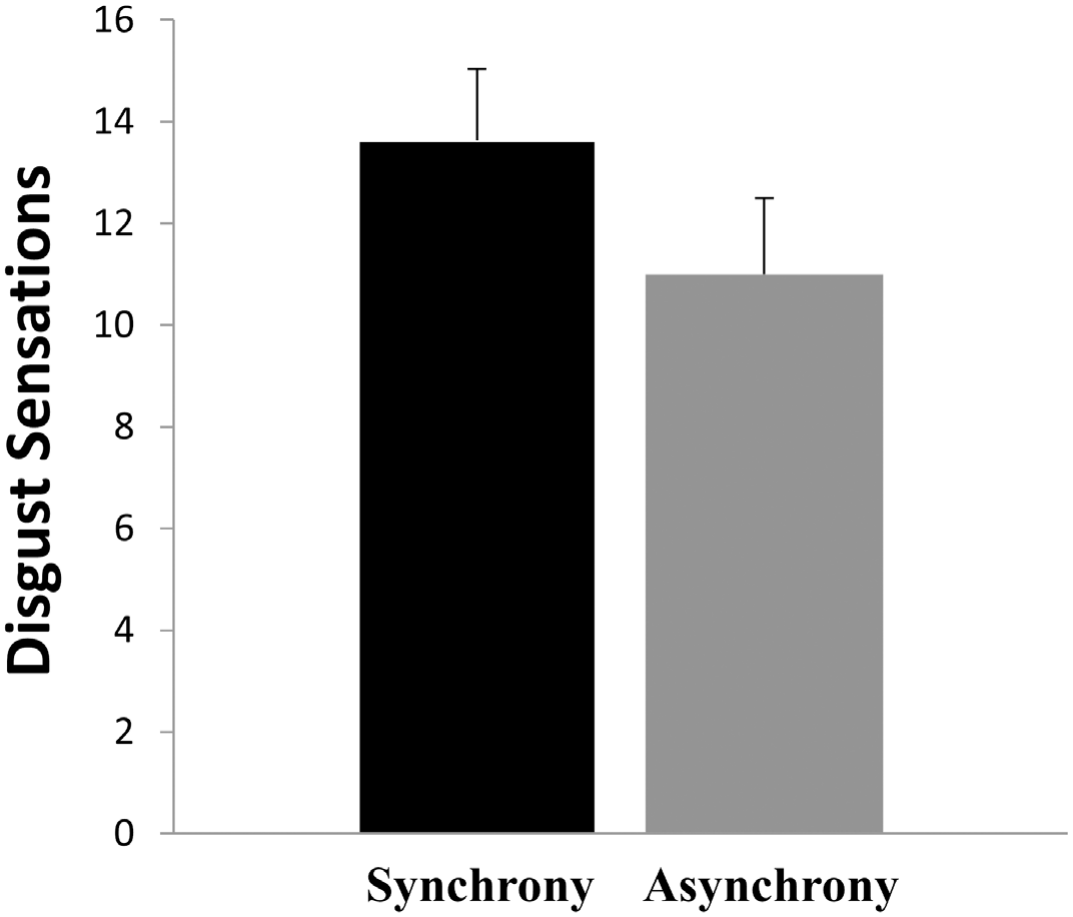
Disgust ratings during the synchronous and the asynchronous condition. (A) Error bars = standard error means.

Nine out of 11 participants reported experiencing greater disgust during the synchronous condition compared to the asynchronous control condition. (One participant reported greater disgust during the asynchronous control condition compared to the synchronous condition, by a single score; one participant reported equal disgust during the two conditions.) On average, participants reported significantly greater disgust during the synchronous condition compared to the asynchronous control condition [*M* = 13.6 (*SD* = 4.6) vs. *M* = 11.0 (*SD* = 5.0)], *N = 11*, *Z* = 2.7, *p* = .004 (one-tailed) (with Bonferroni correction, *p* = .008) (see Fig 3).

### Control experiment

**Fig 4:**
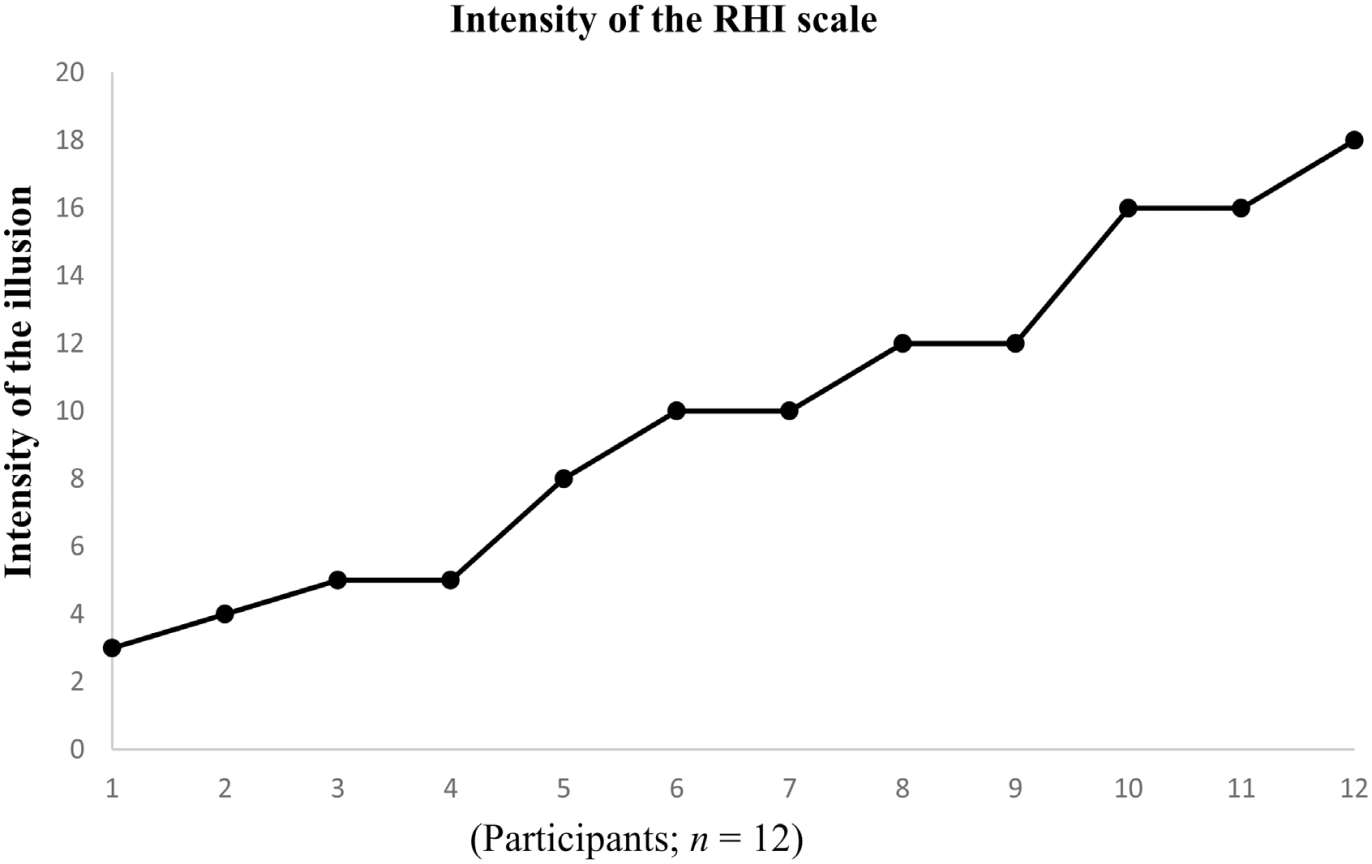
Participants’ ratings on the intensity of the RHI scale for the control experiment.

As expected, the majority of participants in the control group experienced the RHI: i.e., 12 out of 18 participants (67%). The intensity of the illusion ranged from 3–18 (*M* = 9.9, *SD* = 5.1; see Fig 4). [The mean intensity of the RHI did not differ significantly from that of the main experiment (as reported above), *t*(21) = 1.3, *p* = .22.] Six participants were excluded; two participants had a more intense RHI during asynchronous stroking and four participants scored less than 3 out of 20 on the intensity of the RHI scale during synchronous stroking.

Reported “coldness” did not differ during the synchronous condition compared to the asynchronous control condition, when an ice cube was placed on the rubber hand [*M* = 6.1 (*SD* = 3.6) vs. *M* = 6.2 (*SD* = 2.5), *t*(11) = .13, *p* = .90].

## Discussion

In brief, we found that participants reported sensations of disgust arising from a fake hand when a “contaminated” object was placed on it during the RHI. Almost all participants (i.e., 9 out 11) reported experiencing greater disgust during the synchronous condition compared to the asynchronous control condition. And on average, such reported disgust was significantly greater during the synchronous condition compared to the asynchronous control condition.

This study suggests that once the RHI is established, if the fake hand is contaminated, brain centers implicated in disgust (such as the insula, amygdala, and the anterior cingulate cortex) might become activated. The findings argue against a strictly hierarchical modular approach to brain function and suggest that a four-way multisensory interaction occurs between vision, touch, proprioception on the one hand and primal emotions like disgust on the other.

While this study is the first to suggest a link between the RHI and the experiencing of disgust, intriguingly, previous research has shown the involvement of the insula in illusory limb ownership [16, 22, 23]. For example, imaging studies have found activation of the insula in response to the RHI [22]; and when a fake hand was threatened during the illusion [16]. One suggested role of the insula is interoception [24]. And consistent with this, research has found that interoceptive sensitivity is inversely related to the illusion of ownership during the RHI [25]. Interestingly also, a recent study found evidence for a common neural representation in the insula for both interoception and gustation [26]. A more general function of the insula may include processing of self-related information and self-awareness (possibly as a terminal cortical station of interoception and other bodily signals) [24, 27].

In the present control experiment, reported “coldness” did not differ during the synchronous and asynchronous condition. Consistent with this, Mohan et al. found no effect of the RHI on heat or cold pain threshold [20]. Another study found no difference in heat pain threshold between the synchronous and asynchronous stroking of a virtual arm; however, they did find increases in heat pain threshold between the synchronous condition and the viewing of virtual non-corporeal objects and in the absence of virtual reality; suggesting increases in heat pain threshold following ownership of a virtual body [28]. In a related experiment, a virtually reddened arm decreased pain threshold compared with normal and bluish skin [29].

The primary goal of this study was to explore OCD-like disgust and contamination sensations through the RHI, with the aim of potentially informing novel exposure techniques for OCD. While the present study-sample was comprised of healthy volunteers, we believe that the findings might generalize to individuals with clinical contamination-type OCD. In fact, one would expect that such patients would experience even greater disgust and significantly higher levels of anxiety, when the dummy is contaminated during the RHI.

For long, the primary intervention for the treatment of OCD, including contamination aversion, has been exposure therapies: exposing OCD sufferers to contamination [either gradually as in systematic desensitization or more rapidly as in flooding] and preventing compulsive rituals; which in turn help them overcome their fear (e.g., [30]). This therapeutic intervention is commonly referred to as exposure and response prevention (ERP). Our findings raise the possibility of using the RHI to conduct exposures for individuals with contamination-type OCD. Using the RHI might particularly be useful in cases of severe OCD where the patient is reluctant (e.g., is too anxious) to conduct direct exposures; i.e., exposures where “contaminated” objects are in direct contact with the patient’s skin. In such cases, conducting an initial exposure using the RHI might be a useful step (in their “exposure hierarchy”) prior to contaminating the patient’s actual skin. It would thus serve as a “bridge” or “transitional-link” during systematic desensitization. Another possibility is that the RHI could be used in a type of “flooding exposure” approach. For example, it may be that after a number of consecutive trials (say after 10 trials of rapid exposure)—stroking the fake and real hand whilst “contaminating” the fake hand—patients habituate not only to feelings of contamination in the dummy, but also generalize such habituation to their own body; resulting in an overall global reduction in contamination aversion.

Our findings may have clinical implications for other psychiatric disorders. For example, the RHI could be used to conduct exposures for individuals suffering from needle phobia; a condition characterized by severe phobic responses and avoidance of injections (e.g., blood tests) associated with medical care, affecting up to 10% of the general population [31]. Doing “realistic” exposures for this clinical population is difficult given the practical limitations of injecting needles into a patient’s real arm (e.g., multiple times to gain the benefits of the exposure). By contrast, the RHI may conveniently be used to conduct such exposures, by injecting needles into a fake arm, once the dummy arm has been incorporated into the body image.

These findings are preliminary. As noted, participants were not informed that the disgust stimuli were fake, and did not explicitly state that they thought this was so. Nonetheless, we cannot rule out that some participants were in doubt about whether they were real or not, and that this might have affected the results. Future studies should include psychophysical measures [e.g., galvanic skin response (GSR)] and brain imaging to better index disgust sensations. Additional experiments are also needed to prove beyond doubt that the subjects’ rating of disgust in the contaminated hand is not biased by his expectation of what the experimenter might want to hear; e.g. would washing the rubber hand diminish anxiety even though it wouldn’t be “expected'' by the subject or on any rational grounds? Or would the subject’s facial expression [as measured by Electromyography (EMG)] change even when no one was watching? Moreover, the possible novel approaches to exposure mentioned here need further clinical investigation. Since this study did not include a sample of OCD patients, the implications for OCD should be considered speculative until further research has been conducted. We are currently pursuing such clinical studies. Also given that the insula, which mediates disgust in OCD [32], may play a key role in interoception and processing of self [24, 27], future research should examine the relationship between contamination related OCD and body ownership. It is possible that similar to eating disorders [19], which may share a common etiological relationship with OCD [33], OCD too results in greater malleability for the RHI. The experiment we report here raises the possibility of using strictly behavioral approaches toward linking basic concepts and ideas from psychology and cognitive neuroscience with that of psychiatry, possibly paving the way for future therapies.
